# Increased enzymatic hydrolysis of sugarcane bagasse by a novel glucose- and xylose-stimulated β-glucosidase from *Anoxybacillus flavithermus* subsp. *yunnanensis* E13^T^

**DOI:** 10.1186/s12858-017-0079-z

**Published:** 2017-03-16

**Authors:** Yang Liu, Rui Li, Jing Wang, Xiaohan Zhang, Rong Jia, Yi Gao, Hui Peng

**Affiliations:** 1 Anhui Provincial Engineering Technology Research Center of Microorganisms and Biocatalysis. School of Life Sciences, Anhui University, Hefei, Anhui China; 2 College of Biological and Food Engineering, Chuzhou University, Chuzhou, Anhui China

**Keywords:** Conversion of sugarcane bagasse, β-Glucosidase, Glucose-stimulated, Thermostability, Xylose-stimulated

## Abstract

**Background:**

β-Glucosidase is claimed as a key enzyme in cellulose hydrolysis. The cellulosic fibers are usually entrapped with hemicelluloses containing xylose. So there is ongoing interest in searching for glucose- and xylose-stimulated β-glucosidases to increase the efficiency of hydrolysis of cellulosic biomass.

**Results:**

A thermostable β-glucosidase gene (*Bglp*) was cloned from *Anoxybacillus flavithermus* subsp. *yunnanensis* E13^T^ and characterized. Optimal enzyme activity was observed at 60 °C and pH 7.0. Bglp was relatively stable at 60 °C with a 10-h half-life. The kinetic parameters *V*_max_ and *K*_m_ for *p*-nitrophenyl-β-D-glucopyranoside (*p*NPG) were 771 ± 39 μmol/min/mg and 0.29 ± 0.01 mM, respectively. The activity of Bglp is dramatically stimulated by glucose or xylose at concentrations up to 1.4 M. After Bglp was added to Celluclast® 1.5 L, the conversion of sugarcane bagasse was 48.4 ± 0.8%, which was much higher than of Celluclast® 1.5 L alone. Furthermore, Bglp showed obvious advantages in the hydrolysis when initial concentrations of glucose and xylose are high.

**Conclusions:**

The supplementation of BglP significantly enhanced the glucose yield from sugarcane bagasse, especially in the presence of high concentrations of glucose or xylose. Bglp should be a promising candidate for industrial applications.

**Electronic supplementary material:**

The online version of this article (doi:10.1186/s12858-017-0079-z) contains supplementary material, which is available to authorized users.

## Background

The production of biofuels from renewable cellulosic biomass is important for the development of alternative energy. The efficient hydrolysis of cellulose requires the synergistic action of endoglucanases, cellobiohydrolases and β-glucosidases (EC 3.2.1.21). β-Glucosidase is responsible for the hydrolysis of oligosaccharides and cellobiose into glucose. Because oligosaccharides and cellobiose act as strong inhibitors of both endoglucanases and cellobiohydrolases, β-glucosidase is considered as a key enzyme for efficient cellulose hydrolysis [1].

However, the efficient hydrolysis of cellulosic biomass is more difficult than that of pure cellulose, because the cellulosic fibers are usually entrapped in other structural biopolymers, mainly hemicelluloses and lignin. Hemicelluloses consist of an available bulk source of xylose. In this context, glucose- and xylose-stimulated β-glucosidases appear to be particularly well suited to maximize the overall efficiency of hydrolysis of cellulosic biomass, acting in association with endo- and exocellulases and xylanases [2]. Recently, many β-glucosidases with different glucose-tolerant or glucose-stimulated have been reported [3–6]. However, only a few glucose- and xylose-stimulated β-glucosidases have been characterized [7–9]. The advantage of the β-glucosidase group in practical cellulosic biomass hydrolysis is not experimentally studied.

*Anoxybacillus flavithermus* ssp. *yunnanensis* E13^T^ is a thermophilic bacterium that grows at 60 °C [10]. We determined the whole genome sequence of the strain E13^T^ [11] and noted that a gene sequence (KF453503, *BglP*), defined as β-glucosidase in function annotation based on Genbank database, exhibited similarity with some putative β-glucosidases without being biochemically characterized. In this paper, the gene *BglP* was cloned, expressed and characterized in detail. The novel thermostable BglP is a glucose- and xylose-stimulated β-glucosidase. The supplementation of BglP significantly enhanced the glucose yield from sugarcane bagasse, especially in the presence of high concentrations of glucose or xylose.

## Results

### Sequence analysis of Bglp

A β-glucosidase gene (*Bglp*) of 1347 bp with a predicted molecular weight of 52 kDa was revealed by the whole genome sequencing of *A. flavithermus* subsp. *yunnanensis* E13^T^. No signal peptide was found. The amino acid sequence of Bglp showed the highest sequence similarity of 91% with a glucose-tolerant β-glucosidase from *Anoxybacillus* sp. DT3-1 [12], 60% with a β-glucosidase from *Bacillus bogoriensis* (WP_026675503), 56% with a β-glucosidase from *Caldicoprobacter oshimai* (WP_025747479) and 55–57% with some β-glucosidases from *Thermoanaerobacter* sp. strains (ADD25173 and CAA91220). The sequence alignment of Bglp with the homologous is shown in Additional file 1: Figure S1. Two conserved catalytic nucleophile regions (Asn^163^-Glu^164^ and Glu^354^-Asn^355^-Gly^356^) which are highly conserved throughout the glycoside hydrolase family 1 (GH1) are identified in Bglp.

### Enzymatic properties of the purified BglP

The recombinant BglP was expressed in soluble form in *E. coli* BL21 (DE3) cells and purified with Ni-NTA affinity chromatography. The purified BglP was a single band on 10% SDS-PAGE (Additional file 1: Figure S2). The apparent molecular mass of BglP was approximately 52 kDa, which corresponded to the calculated mass.

**Fig. 1:**
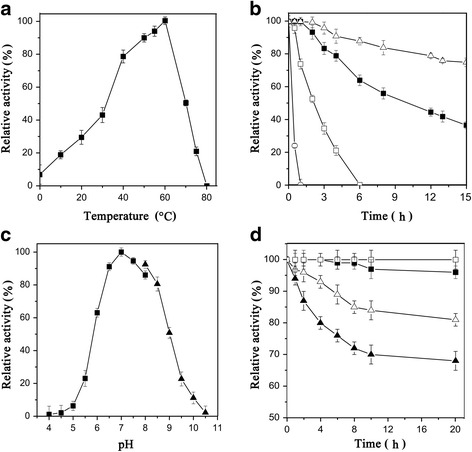
Effects of temperature and pH on β-glucosidase activity and stability of BglP. **a** Effect of temperature on enzyme activity. **b** Effect of temperature on the stability of BglP. The enzyme solution was incubated in Na_2_HPO_4_-citric acid buffer (pH 7.0, without substrate) at 55 (∆), 60 (■), 65 (□) and 70 °C (○) for different time periods, and the residual activity was measured. **c** Effect of pH on enzyme activity. The ability was assayed at 60 °C in different buffers: 50 mM Na_2_HPO_4_-citric acid buffer pH 4.0 to 8.0 and 50 mM Glycine-NaOH buffer pH 8.0 to 10.0. **d** Effect of pH on stability of BglP. The enzyme was incubated at 25 °C in each buffer pH 6.0 (▲), pH 7.0 (∆), pH 8.0 (■) and pH 9.0 (□) for different time periods, and the residual activity was measured. Values are the means ± SD of six experiments (*n* = 6)

The recombinant BglP exhibited the maximum activity at 60 °C (Fig. 1a), and it exhibited 50% of its optimal activity at 70 °C. The temperature stability of BglP was assayed between 55 and 70 °C up to 15 h (Fig. 1b). BglP showed good stability at the optimum temperature 60 °C, as the half-life at 60 °C was approximately 10 h. BglP exhibited optimal activity at pH 7.0, with more than 80% enzyme activity at pH from 7.0 to 8.5 (Fig. 1c). The enzyme was found to be very stable in the range of 7.0–9.0 (Fig. 1d). BglP retained over 81% of initial activity after 20 h of incubation at pH 7.0 and it was totally stable at pH 8.0 and 9.0 (Fig. 1d). The activity of BglP toward various substrates was measured to determine substrate specificity (Table 1). The maximum activity was obtained with *p*NPG (842 ± 15.5 U/mg). The *K*_m_ and the *V*_max_ were 0.29 ± 0.01 mM and 771 ± 39 μmol/min/mg protein, respectively, against *p*NPG (Additional file 1: Figure S3a). *p*-Nitrophenyl-β-D-lactopyranoside, *p*-nitrophenyl-β-D-fucopyranoside and *p*-nitrophenyl-β-D-galactoside (*p*NPGal) were hydrolyzed at approximately 15, 13 and 8%, respectively, of the activity observed with *p*NPG. The activities of BglP for *p*NPG and *p*NPGal that contained β-1-4 linkages were much higher than for *o*NPG and *o*NPGal that contained β-1-2 linkages. BglP was able to hydrolyze all of the cello-oligosaccharides tested: cellobiose, cellotriose, cellotetraose and cellopentaose. The *K*_m_ and the *V*_max_ for cellobiose were 12.3 ± 0.7 mM and 107 ± 8.5 μmol/min/mg, respectively (Additional file 1: Figure S3b). It had activity toward lactose and laminaribiose which has β-1, 3-linked galactose.

**Table 1:** Hydrolysis activities of BglP on various substrates

Substrate^a^	Linkage of glycosyl group	Relative activity^b^ (%)
*p*-Nitrophenyl-β-D-glucopyranoside (*p*NPG)	(β-1,4) Glucose	100
*o*-Nitrophenyl-β-D-glucopyranoside (*o*NPG)	(β-1,2) Glucose	51 ± 1.6
*p*-Nitrophenyl-β-D-lactopyranoside (*p*NPLac)	(β-1,4) Lactose	15.3 ± 0.6
*p*-Nitrophenyl-β-D-fucopyranoside (*p*NPFuc)	(β-1,4) Fucose	13.2 ± 0.4
*p*-Nitrophenyl-β-D-galactoside (*p*NPGal)	(β-1,4) Galactose	8.2 ± 0.2
*o*-Nitrophenyl-β-D-galactoside (*o*NPGal)	(β-1,2) Galactose	2.0 ± 0.3
Cellobiose	(β-1,4) Glucose	17.5 ± 0.4
Cellotriose	(β-1,4) Glucose	7.9 ± 0.1
Cellotetraose	(β-1,4) Glucose	6.7 ± 0.4
Cellopentaose	(β-1,4) Glucose	4.9 ± 0.2
Lactose	(β-1,4) Galactose	11.0 ± 0.6
Laminaribiose	(β-1,3) Glucose	5.6 ± 0.2

^a^No activity or poorly activity was detected with *p*-Nitrophenyl-α-D-glucopyranoside, *p*-nitrophenyl-β-D-xyloside, CMC and Avicel®

^b^The relative activity of the most preferentially hydrolyzed substrate *p*NPG was taken as 100%. Values are the means ± SD of six experiments (*n* = 6)

The influences of different metal ions and reagents on the BglP activity were investigated (Table 2). When the concentrations of metal ions were less than 5 mM, the activity was not significantly inhibited, retaining above 77% residual activity. When the concentrations increased up to 10 mM, less than 50% residual activities were observed in the presence of CuCl_2_ and FeCl_2_, while ZnCl_2_, FeCl_3_, CoCl_2_, MnCl_2_, CaCl_2_ and MgCl_2_ had slightly inhibitory effect. The addition of most reagents also had slight effect on enzymatic activity except SDS (Table 2), and BglP displayed more than 55% original activity. The results showed that BglP was insensitive to many metal ions and reagents, and did not require metals as co-factors.

**Table 2:** The effects of various metal ions and reagents on the activity of BglP

Substances	Relative activity (%)	
	5 mM	10 mM
Control	100	100
MgCl_2_	101 ± 1.3	102 ± 1.2
CaCl_2_	99 ± 0.7	96 ± 0.5
MnCl_2_	95 ± 1.6	92 ± 1.3
FeCl_3_	98 ± 1.0	92 ± 0.9
CoCl_2_	99 ± 0.5	86 ± 1.6
ZnCl_2_	98 ± 1.0	83 ± 0.4
CuCl_2_	91 ± 1.4	44 ± 0.5
FeCl_2_	77 ± 0.8	20 ± 0.6
DTT	99 ± 0.5	98 ± 0.9
Urea	98 ± 1.5	96 ± 1.3
EDTA	96 ± 0.9	82 ± 1.4
β-Mercaptoethanol	99 ± 1.2	92 ± 1.6
DMSO^a^	5%	10%
	97 ± 0.7	90 ± 1.6
Triton X-100^a^	1%	2%
	91 ± 1.0	80 ± 0.5
Tween 80^a^	82 ± 0.9	73 ± 1.4
SDS	3.4 ± 0.4	*ND*

The enzymes were pre-incubated for 30 min at 60 °C with each additive before activity measurement with *p*NPG as substrate. The enzyme activity of BglP without metal ions was taken as 100% (842 ± 15.5 U/mg)

*ND* not determined. Values are the means ± SD of six experiments (*n* = 6)

^a^The concentrations was percent volume by volume (*v*/*v*)

### Effect of sugars on the activity and thermostability of BglP

**Fig. 2:**
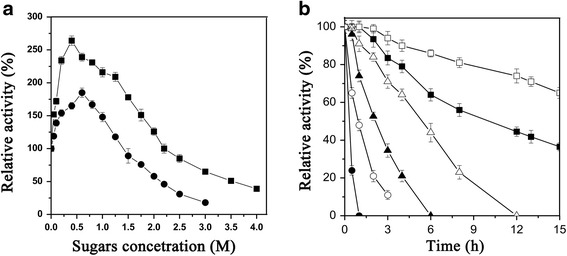
**a** Stimulatory effects of glucose and xylose on the activity of BglP. Hydrolytic activity on 5 mM *p*NPG in the presence of increasing concentrations of glucose (■) or xylose (●). **b** Effect of glucose on thermostability of BglP. The enzyme were incubated at 60 °C in the presence (1 M, □) or absence (control, ■) of glucose, at 65 °C in the presence (1 M, ∆) or absence (control,▲) of glucose, and at 70 °C in the presence (1 M, ○) or absence (control, ●) of glucose. One hundred percent specific activity corresponded to 842 ± 15.5 U/mg, estimated in same conditions without any additional sugar. Values are the means ± SD of six experiments (*n* = 6)

The effects of various sugars (100 mM each of glucose, xylose, galactose, arabinose, mannose, maltose, fructose, sucrose, cellobiose and ribose) on BglP activity were determined (Additional file 1: Table S1). Surprisingly, all of the 11 tested sugars showed no significant inhibition of BglP. The BglP activity was significantly stimulated by glucose and xylose, respectively. Therefore, the activity on different concentrations of the two sugars was further determined (Fig. 2a). A maximal 2.6-fold stimulation by glucose was observed at 0.4 M, and a maximal 1.8-fold stimulation by xylose was found at 0.6 M. BglP activated by glucose and xylose was unusually remained at concentrations up to 2.2 and 1.4 M, respectively. At fixed glucose concentrations of 0.4 M, increasing concentrations of *p*NPG (0.05–1.6 mM) stimulated the enzymatic activity. The *K*_m_ and *V*_max_ were 0.37 ± 0.02 mM and 1550 ± 101 μmol/min/mg, respectively. At fixed xylose concentrations of 0.6 M, the *K*_m_ and *V*_max_ calculated from the stimulation of activity were 0.48 ± 0.03 mM and 1333 ± 86 μmol/min/mg protein, respectively (Additional file 1: Figure S4). When glucose and xylose was further increased, the enzyme activity was gradually inhibited, with inhibition constant (*K*_i_) values of 3.8 ± 0.22 and 2.0 ± 0.15 M, respectively (Additional file 1: Figure S5).

To fully exploit the effect of glucose on BglP, the enzymatic thermostability at 60-70 °C was determined in the presence of a final concentration of 1 M glucose (Fig. 2b). The result suggested that the stability significantly increased in the presence of glucose. The BglP preserved 65% of its original activity after 15-h incubation at 60 °C in the presence of glucose. The half-life of BglP at 65 °C was approximately 2 h, while the half-life prolonged to approximately 5.5 h in the presence of glucose.

### Conversion of sugarcane bagasse under high glucose and xylose concentrations

**Fig. 3:**
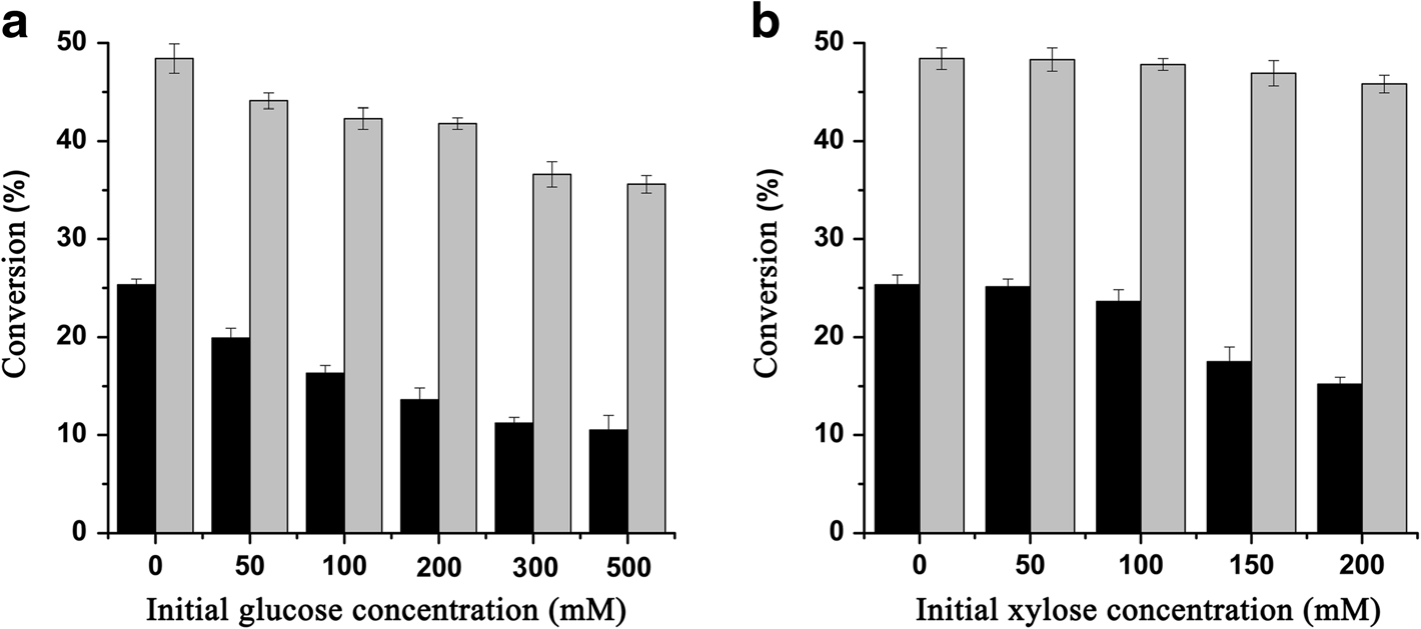
Effects of glucose (**a**) and xylose (**b**) on the conversions of sugarcane bagasse. The reactions were performed at 60 °C in 50 mM Na_2_HPO_4_-citric acid buffer (pH 7.0). The concentration of the substrate was 10% (*w/v*). Addition of BglP to Celluclast® 1.5 L significantly improved the conversions under all the conditions tested. Celluclast® 1.5 alone: black bars; BglP and Celluclast® 1.5 L: grey bars. Values are the means ± SD of six experiments (*n* = 6)

To evaluate the application potential of BglP in the cellulosic biomass hydrolysis where glucose and xylose concentrations are high, the conversion of sugarcane bagasse by Celluclast® 1.5 L with or without BglP was performed in the presence of various initial concentrations of glucose (50–500 mM) and xylose (50–200 mM) (Fig. 3). The cellulose content was 41.7% (*w/w*) in the sugarcane bagasse. After a 48-h hydrolysis at 60 °C, pH 7.0, the concentration of glucose released by Celluclast® 1.5 L alone was measured to be 10.5 ± 0.7 mg/ml, representing 25.3 ± 0.5% of the conversion. When the synergistic action of BglP and Celluclast® 1.5 L was performed, a 1.9-fold conversion increase was observed, and the final conversion was up to 48.4 ± 0.8% (Fig. 3).

The conversion of sugarcane bagasse decreased to 19.9 ± 0.8% (Celluclast® 1.5 L alone) in the presence of 50 mM of glucose in the initial reaction mixture. The conversions further decreased with increasing initial concentrations of glucose. The lowest 10.5 ± 0.1% of conversion was observed at 500 mM initial glucose. The β-glucosidase in Celluclast® 1.5 L showed a lower hydrolytic efficiency in the presence of high concentrations of glucose. The supplementation of BglP significantly enhanced the conversion under all the concentrations of glucose. The conversion of the synergistic action at 500 mM was 35.6 ± 0.5% (Fig. 3a), which was 3.4-fold higher than that of Celluclast® 1.5 L alone.

Although the addition of 50 mM xylose had no effect on the conversion that performed by Celluclast® 1.5 L alone, a significant inhibition was observed when the xylose concentration was over 100 mM (Fig. 3b), which suggested the β-glucosidase in Celluclast® 1.5 L is only sensitive to high concentrations of xylose. The conversion by Celluclast® 1.5 L alone at 200 mM decreased to 15.2 ± 0.7%. When BglP was added to the reaction mixture, the conversion only weakly decreased from 48.4 ± 0.8 to 45.8 ± 0.9% with increasing initial concentrations of xylose from 0 to 200 mM. The conversion at 200 mM was 3.0-fold higher than that of Celluclast® 1.5 L alone.

## Discussion

β-Glucosidase is an important component of the cellulose enzyme system. A strong inhibition of β-glucosidase by glucose is most commonly observed [1]. Searching for novel β-glucosidases with insensitive to glucose attracts more interest in recent years. Based on the different influences of glucose on the activity, Cao et al. [13] concluded that β-glucosidases can be divided roughly into three groups: (1) the glucose-sensitive β-glucosidases; (2) the glucose-tolerant β-glucosidases that are not activated by glucose; (3) the glucose-stimulated β-glucosidases. The glucose-stimulated β-glucosidases are less common, as compared with two previous groups. The BglP activity is stimulated 2.6-fold by 0.4 M glucose (Fig. 2a), therefore BglP should belong to the last group.

The stimulatory specific activity of BglP was as high as 2163 ± 11.2 U/mg at 0.4 M glucose, and this level was higher than most of glucose-stimulated β-glucosidases. The β-glucosidases from uncultured bacterium [13, 14], *Thermoanaerobacterium aotearoense* [15], *Anoxybacillus* sp. DT3-1 [12] and *Neotermes koshunensis* [6] were stimulated about 4.0-, 2.7-, 1.4-, 1.3- and 1.3-fold by glucose, respectively and the stimulatory specific activity were about 11, 10.8, 145, 1505 and 16 U/mg, respectively. One of the reasons for the stimulation could be transglycosylation [6, 16]. Therefore, we analyzed the reaction products of BglP after incubation with 10 mM *p*NPG and glucose (100, 250 and 500 mM, respectively). No other product except glucose was determined, suggesting a lack of transglycosylation activity in BglP. Souza et al. [2] speculated that the stimulatory effect of glucose may be attributed to its binding to modulator binding sites, probably inducing conformational changes that stimulate the hydrolyzing activity. Further work will be necessary to determine whether the same applies to BglP.

Besides glucose stimulation, BglP is also stimulated by xylose, which distinguishes the enzyme from the stimulatory group of β-glucosidases. Xylan chains are entrapped inside cellulosic fibers. During cellulose hydrolysis, the final result of synergistic action of xylanases and cellulases is a high product of both glucose and xylose [2]. Thus, the glucose- and xylose-stimulated β-glucosidases are very attractive to improve the effectiveness of cellulose hydrolysis. However, to our knowledge, only six β-glucosidases were reported to be stimulated by both glucose and xylose (Table 3). The practical hydrolysis of cellulosic biomass with the enzymes has not been done so far. Compared with these β-glucosidases, BglP displayed higher stimulation/tolerance levels, stronger specific activity and better thermostability. Therefore, its synergistic act with Celluclast® 1.5 L on converting sugarcane bagasse was investigated under the optimum conditions of BglP in the presence/absence of glucose or xylose (Fig. 3).

**Table 3:** Characteristics of BglP from *A. flavithermus* subsp. *yunnanensis* E13^T^ and other glucose- and xylose-stimulated β-glucosidases

Origins	Relative activity (%) ^a^		Inhibitory concentration (M)	Specific activity (*p*NPG, U/mg)	Optimal temperature	Thermostability (Half-life)	Source of strain	Reference
	Glucose	Xylose						
*A. flavithermus* subsp. *yunnanensis*	258 ± 5.1	182 ± 3.4	2.2 and 1.4 for glucose and xylose, respectively	842 ± 15.5	60	~10 h at 60 °C;	Bacterium	Present work
*S. thermophilum*	204 ± 12	191 ± 14	0.7 and 0.5 for glucose and xylose, respectively	8.9	60	20 min at 55 °C	Fungi	[8]
*H. insolens*	180 ± 9	200 ± 11	0.4	36.4	60	44 min at 55 °C	Fungi	[7]
*H. grisea* var. *thermoidea*	188	202	0.5	29.5	50	7 min at 60 °C	Fungi	[23]
*B. halodurans*	135.0 ± 4.3	131.0 ± 0.2	~0.8-0.9	93	50	30 min at 50 °C	Bacterium	[24]
Uncultured bacterium	120	150	0.3 for glucose	183.9	50	*ND*	Soil metagenome	[5]
*Marinomonas* sp. MWYL1	~132	~145	0.6	389	40	~11 min at 45 °C	Bacterium	[9]

*ND* not determined

^a^One hundred percent specific activity (control) was estimated in the absence of carbohydrates

In the case without initial sugar, the conversion of sugarcane bagasse by BglP was improved 1.9-fold than of Celluclast® 1.5 L alone. The supplement of β-glucosidases from a metagenomic library [13] and *Thermoanaerobacterium aotearoense* [15] to the conversion of sugarcane bagasse resulted in about 1.5- and 1.2-fold enhancement, respectively. The enhancement levels were lower than that of BglP, which may be due to the higher specific activity of BglP. In the case with initial sugar, since the β-glucosidases in Celluclast® 1.5 L is sensitive to both glucose and xylose, the advantages of BglP were more obvious. The conversion supplemented with BglP was 3.4- and 3.0-fold higher than that of Celluclast® 1.5 L alone at 500 mM of initial glucose and 200 mM of initial xylose, respectively. The results not only clearly revealed the significant role of the glucose- and xylose-stimulated β-glucosidases in the practical hydrolysis of cellulosic biomass, but also showed that BglP should have important practical implication in cellulose refining.

## Conclusions

BglP from *A. flavithermus* subsp. *yunnanensis* E13^T^ is a thermostable β-glucosidase, whose hydrolysis rate (*V*_max_ value) is very fast. The activity of Bglp is dramatically stimulated by glucose and xylose, and has high tolerance to glucose and xylose. The supplement of Bglp to the hydrolysis mixture of sugarcane bagasse containing Celluclast® 1.5 L resulted in great enhancement of the conversion, especially in the presence of high concentrations of glucose and xylose.

## Methods

### Bioinformatics analysis

Homologues of AmyP were obtained by searching the NCBI protein database using the BLAST tools [17]. Sequence alignments were performed using the ClustalW [18]. BLAST analyses suggested that Bglp belongs to GH1. A GH1 domain (pfam00232) was served as definition criteria for conserved nucleophile regions.

### Cloning, expression and purification of β-glucosidase BglP

The strain *A. flavithermus* ssp. *yunnanensis* E13^T^ was isolated from a hot spring in our previous study [10]. It is available from the China Center for Type Culture Collection (AB2010187^T^) and the Korean Collection for Type Cultures (13759^T^). The β-glucosidase gene *BglP* was amplified by PCR using the primers (see Additional file 1). The PCR product was digested and ligated into pET22b, and then the plasmid pET22b-*Bglp* was transformed into *E. coli* BL21 (DE3) (Novagen). The recombinant BglP was induced by adding a final concentration of 0.8 mM isopropyl-β-D-thiogalactopyranoside (IPTG) at OD_600_ about 0.5–0.6, and incubated further for 5 h. The cells were harvested and lysed by sonication. The lysate was heat treated at 60 °C for 30 min to denature the thermolabile proteins in *E. coli*, and then centrifuged (20,000 × g, 30 min). The supernatant was loaded on a Ni-NTA agarose gel column for purification. The protein was analyzed by sodium dodecyl sulfate-polyacrylamide gel electrophoresis (SDS-PAGE). The protein concentration was determined according to Bradford method.

### Enzyme assay

β-Glucosidase activity was determined with *p*-nitrophenyl-β-D-glucopyranoside (*p*NPG, Sigma) as a substrate. The reaction mixture contained 10 μl of enzyme and 290 μl of 50 mM Na_2_HPO_4_-citric acid buffer (pH 7.0) with 5 mM *p*NPG. The reaction was incubated at 60 °C for 10 min and stopped by adding 1 ml of 1 M Na_2_CO_3_. The *p*-nitrophenol (*p*NP) released was measured at *A*_410_. Other aryl-glycoside substrates were assayed under the same conditions. BglP activity against various saccharides was estimated with 1% substrate concentration, and the glucose released was quantified by a glucose oxidase-peroxidase assay (Sangon, China). The hydrolysis of carboxymethyl cellulose (CMC) and microcrystalline cellulose (Avicel®) was measured after 30 min reaction, employing 20.0 U of *p*NPG activity. The reducing sugar released was determined according to the classical method [19]. In all analyses, 1 U was defined as the amount of enzyme that releases 1 μmol of product per min under the assay conditions. All experiments were performed at least three times and three independent reproductions were carried out.

### Properties of recombinant BglP

The optimal temperature was measured over a temperature range from 0 to 80 °C at pH 7.0 (50 mM, Na_2_HPO_4_-citric acid buffer). Thermostability was determined by incubated the protein at 55–70 °C during 0.5 - 15 h. The residual activities were determined in standard reaction condition. The optimum pH of BglP was determined at 60 °C. To measure the pH stability, the purified BglP was incubated at pH 6.0–9.0 at 25 °C. Samples were removed at varying time intervals, and the residual activities were measured.

The influence of various metal ions (5 and 10 mM) on the BglP activity was investigated using MgCl_2_, CaCl_2_, FeCl_3_, CoCl_2_, ZnCl_2_, MnCl_2_, CuCl_2_ and FeCl_2_. The effects of dithiothreitol (DTT), urea, ethylenediaminetetraacetic acid (EDTA), β-mercaptoethanol, dimethyl sulfoxide (DMSO), Triton X-100, Tween 80 and sodium dodecyl sulfate (SDS) were determined. All enzyme activity was determined after pre-incubation of the purified BglP with above compounds in at 60 °C for 30 min. The BglP activity without any additive was taken as a percentage of the activity.

### Determination of kinetic parameters

The Michaelis-Menten constants (*K*_m_) and maximum velocities (*V*_max_) were determined from Lineweaver-Burk plots by using *p*NPG or cellobiose as substrates. The purified BglP was incubated in Na_2_HPO_4_-citric acid buffer (pH 7.0) with the substrates in concentrations ranging from 0.05 to1.6 mM of *p*NPG or 5–70.0 mM of cellobiose at 60 °C. The kinetic parameters of *p*NPG in the presence of 0.4 M of glucose or 0.6 M xylose were established for the stimulation of activity.

The inhibition constants (*K*_i_) for the *p*NPG hydrolysis inhibition by glucose and xylose were estimated using Dixon plots. The concentration ranges of glucose and xylose were 3.0–3.7 and 1.8–2.3 M, respectively. Two concentrations of *p*NPG (5 and 8 mM) were used. All experimental kinetic curves were repeated three times, and three independent reproductions were carried out. The kinetic parameters are given as the mean ± SD of the values calculated for six different experiments (*n* = 6).

### Effects of sugars on BglP activity and stability

The effects of various sugars (100 mM each of glucose, xylose, galactose, arabinose, mannose, maltose, fructose, sucrose, cellobiose and ribose) on the BglP activity were studied. The stimulatory effects of glucose and xylose on the activity were further tested using varying concentrations of glucose (0.05–4.0 M) and xylose (0.05–3.0 M). The BglP was pre-incubated with sugars at 14 °C for 24 h. The residual activities were quantified, and the control activity without sugar was taken as a percentage of the activity.

In determination of the effect of glucose on thermostability, glucose (1 M) was added into the reaction solution and incubated at different temperatures (60, 65 and 70 °C) for different time (0.5–15 h), and then the residual activities were determined.

### Conversion of sugarcane bagasse by BglP

Sugarcane bagasse was preformed according to the previous study [20]. In brief, the sugarcane bagasse (Jing Hui Sugar Group Co. Ltd, China) pretreated with 1% (*v/v*) H_2_SO_4_ with a solid to liquid ratio of 1:20. The solids were carried out in an autoclave at 120 °C for 70 min by using 4% NaOH. Then the alkaline solids were washed with water using until pH changed to 7.0. The dried solids were used for subsequent experiments.

Enzymatic conversion of sugarcane bagasse was performed with 10% (*w/v*, dry basis) substrate concentration in 20 ml of Na_2_HPO_4_-citric acid buffer (pH 7.0). The commercial Cellulast® 1.5 L (Novozymes, Denmark) load was 40 filter paper unite (FPU) per gram of sugarcane bagasse according to the previous study [20]. The purified BglP load was 50 μg per gram of sugarcane bagasse. The flasks with the reaction mixture were carried out in a rotary shaker at 60 °C and 120 rpm. After a 48-h hydrolysis, the glucose released was quantified by HPLC according to method of Lei et al. [21]. The conversion of sugarcane bagasse was calculated according to Cao et al. [13]. In determination of the effects of glucose and xylose on the hydrolysis of sugarcane bagasse, glucose (the final concentration of 50–500 mM) or xylose (50–200 mM) was added into the reaction mixture. A final concentration of 0.2% (*v/v*) toluene was used to prevent microbial contamination during the conversion [22].

## Additional file

Additional file 1: Sequences of primers used for PCR. **Figure S1.** Sequence alignment of Bglp with six similar β-glucosidases. **Figure S2.** SDS-PAGE analysis of recombinant BglP. Kinetic Parameters: **Figure S3.** Effects of various concentrations of pNPG (a) and cellobiose (b) on the activity of recombinant BglP. **Figure S4.** Lineweaver-Burk plots of pNPG hydrolysis by the recombinant BglP in the absence addition (■) and presence of fixed concentrations of glucose (●) or xylose (▲). Inhibitory kinetics. **Figure S5.** Dixon plots of inhibitory effects of glucose (a) and xylose (b) on pNPG hydrolysis by recombinant BglP. **Table S1.** Effect of sugar additions on the BglP activity. (DOC 9253 kb)

## Abbreviations

CMC: Carboxymethyl cellulose
DTT: Dithiothreitol
EDTA: Ethylenediaminetetraacetic acid
GH1: Glycoside hydrolase family 1
IPTG: Isopropyl-β-D-thiogalactopyranoside
*K*_i_: Inhibition constants
*K*_m_: Michaelis-Menten constants
*p*NP: *p*-nitrophenol
*p*NPG: *p*-nitrophenyl-β-D-glucopyranoside
*p*NPGal: *p*-nitrophenyl-β-D-galactoside
SDS: Sodium dodecyl sulfate
SDS-PAGE: Sodium dodecyl sulfate-polyacrylamide gel electrophoresis
*V*_max_: Maximum velocities

## References

[1] Bhatia Y, Mishra S, Bisaria VS (2002). Microbial beta-glucosidases: cloning, properties, and applications. Crit Rev Biotechnol.

[2] Souza FHM, Inocentes RF, Ward RJ, Jorge JA, Furriel RPM (2013). Glucose and xylose stimulation of a β-glucosidase from the thermophilic fungus *Humicola insolens*: A kinetic and biophysical study. J Mol Catal B Enzym.

[3] Li G, Jiang Y, Fan XJ, Liu YH (2012). Molecular cloning and characterization of a novel beta-glucosidase with high hydrolyzing ability for soybean isoflavone glycosides and glucose-tolerance from soil metagenomic library. Bioresour Technol.

[4] Pei JJ, Pang Q, Zhao L, Fan S, Shi H (2012). *Thermoanaerobacterium thermosaccharolyticum* β-glucosidase: a glucose-tolerant enzyme with high specific activity for cellobiose. Biotechnol Biofuels.

[5] Lu J, Du L, Wei Y, Hu Y, Huang R (2013). Expression and characterization of a novel highly glucose-tolerant β-glucosidase from a soil metagenome. Acta Biochim Biophys Sin (Shanghai).

[6] Uchima CA, Tokuda G, Watanabe H, Kitamoto K, Arioka M (2011). Heterologous expression and characterization of a glucose-stimulated β-glucosidase from the termite Neotermes koshunensis in *Aspergillus oryzae*. Appl Microbiol Biotechnol.

[7] Souza FHM, Nascimento CV, Rosa JC, Masui DC, Leone FA, Jorge JA, Furriel RPM (2010). Purification and biochemical characterization of a mycelial glucose- and xylose-stimulated β-glucosidase from the thermophilic fungus *Humicola insolens*. Process Biochem.

[8] Zanoelo FF, de Polizeli ML, Terenzi HF, Jorge JA (2004). Beta-glucosidase activity from the thermophilic fungus *Scytalidium thermophilum* is stimulated by glucose and xylose. FEMS Microbiol Lett.

[9] Zhao W, Peng R, Xiong A, Fu X, Tian Y, Yao Q (2012). Expression and characterization of a cold-active and xylose-stimulated β-glucosidase from *Marinomonas* MWYL1 in Escherichia coli. Mol Biol Rep.

[10] Gao Y, Dai J, Peng H, Liu Y, Xu T (2011). Isolation and characterization of a novel organic solvent-tolerant *Anoxybacillus* sp. PGDY12, a thermophilic Gram-positive bacterium. J Appl Microbiol.

[11] Wang Y, Zheng Y, Wang M, Gao Y, Xiao YZ, Peng H (2014). Non-contiguous finished genome sequence of *Anoxybacillus flavithermus* subsp. *yunnanensis* type strain (E13T), a strictly thermophilic and organic solvent-tolerant bacterium. Stand Genomic Sci.

[12] Chan CS, Sin LL, Chan KG, Shamsir MS, Manan FA, Sani RK, Goh KM (2016). Characterization of a glucose-tolerant β-glucosidase from *Anoxybacillus* sp. DT3-1. Biotechnol Biofuels.

[13] Cao LC, Wang ZW, Ren GH, Kong W, Li L, Xie W, Liu YH (2015). Engineering a novel glucose-tolerant β-glucosidase as supplementation to enhance the hydrolysis of sugarcane bagasse at high glucose concentration. Biotechnol Biofuels.

[14] Biver S, Stroobants A, Portetelle D, Vandenbol M (2014). Two promising alkaline β-glucosidases isolated by functional metagenomics from agricultural soil, including one showing high tolerance towards harsh detergents, oxidants and glucose. J Ind Microbiol Biotechnol.

[15] Yang F, Yang XF, Li Z, Du CY, Wang JF, Li S (2015). Overexpression and characterization of a glucose-tolerant β-glucosidase from T. aotearoense with high specific activity for cellobiose. Appl Microbiol Biotechnol.

[16] Uchiyama T, Miyazaki K, Yaoi K (2013). Characterization of a novel beta-glucosidase from a compost microbial metagenome with strong transglycosylation activity. J Biol Chem.

[17] Altschul SF, Madden TL, Schäffer AA, Zhang J, Zhang Z, Miller W, Lipman DJ (1997). Gapped BLAST and PSI-BLAST: a new generation of protein database search programs. Nucleic Acids Res.

[18] Thompson JD, Gibson TJ, Plewniak F, Jeanmougin F, Higgins DG (1997). The ClustalX windows interface: flexible strategies for multiple sequence alignment aided by quality analysis tools. Nucleic Acids Res.

[19] Miller GL (1959). Use of dinitrosalicylic acid reagent for determination of reducing sugar. Anal Chem.

[20] Borges DG, Junior AB, Farinas CS, Giordano RLC, Tardioli PW (2014). Enhanced saccharification of sugarcane bagasse using soluble cellulase supplemented with immobilized β-glucosidase. Bioresour Technol.

[21] Lei Y, Peng H, Wang Y, Liu YT, Han F, Xiao YZ, Gao Y (2012). Preferential and rapid degradation of raw rice starch by an α-amylase of glycoside hydrolase subfamily GH13_37. Appl Microbiol Biotechnol.

[22] Hamilton LM, Kelly CT, Fogarty WM (1998). Raw starch degradation by the non-raw starch-adsorbing bacterial alpha amylase of Bacillus sp. IMD 434. Carbohydr Res.

[23] Nascimento CV, Souza FH, Masui DC, Leone FA, Peralta RM, Jorge JA, Furriel RPM (2010). Purification and biochemical properties of a glucose-stimulated β-D-glucosidase produced by Humicola grisea var. thermoidea grown on sugarcane bagasse. J Microbiol.

[24] Xu H, Xiong AS, Zhao W, Tian YS, Peng RH, Chen JM, Yao QH (2011). Characterization of a glucose-, xylose-, sucrose-, and galactose-stimulated β-glucosidase from the alkalophilic bacterium *Bacillus halodurans* C-125. Curr Microbiol.

