# Supplementary material for: Increased enzymatic hydrolysis of sugarcane bagasse by a novel glucose- and xylose-stimulated β-glucosidase from Anoxybacillus flavithermus subsp. yunnanensis E13T
Source: BMC Biochem. 2017 Mar 16;18:4. doi: 10.1186/s12858-017-0079-z (PMC5356265; doi:10.1186/s12858-017-0079-z)
Supplement: Additional file 1: — Sequences of primers used for PCR. Figure S1. Sequence alignment of Bglp with six similar β-glucosidases. Figure S2. SDS-PAGE analysis of recombinant BglP. Kinetic Parameters: Figure S3. Effects of various concentrations of pNPG (a) and cellobiose (b) on the activity of recombinant BglP. Figure S4. Lineweaver-Burk plots of pNPG hydrolysis by the recombinant BglP in the absence addition (■) and presence of fixed concentrations of glucose (●) or xylose (▲). Inhibitory kinetics. Figure S5. Dixon plots of inhibitory effects of glucose (a) and xylose (b) on pNPG hydrolysis by recombinant BglP. Table S1. Effect of sugar additions on the BglP activity. (DOC 9253 kb) [file 12858_2017_79_MOESM1_ESM.doc]

**Increased enzymatic hydrolysis of sugarcane bagasse by a novel glucose- and xylose-stimulated β-glucosidase from** ***Anoxybacillus flavithermus* subsp. *yunnanensis*** **E13^T^**

1, Sequences of primers used for PCR:

TTCCATATGCTTCAGTTTCCGAAA (*Nde*I digestion site underlined);

CCCGAGCTCTTTAACTCCATGATTCATG (*Xho*I digestion site underlined).

2, Sequence alignment of Bglp with six similar β-glucosidases


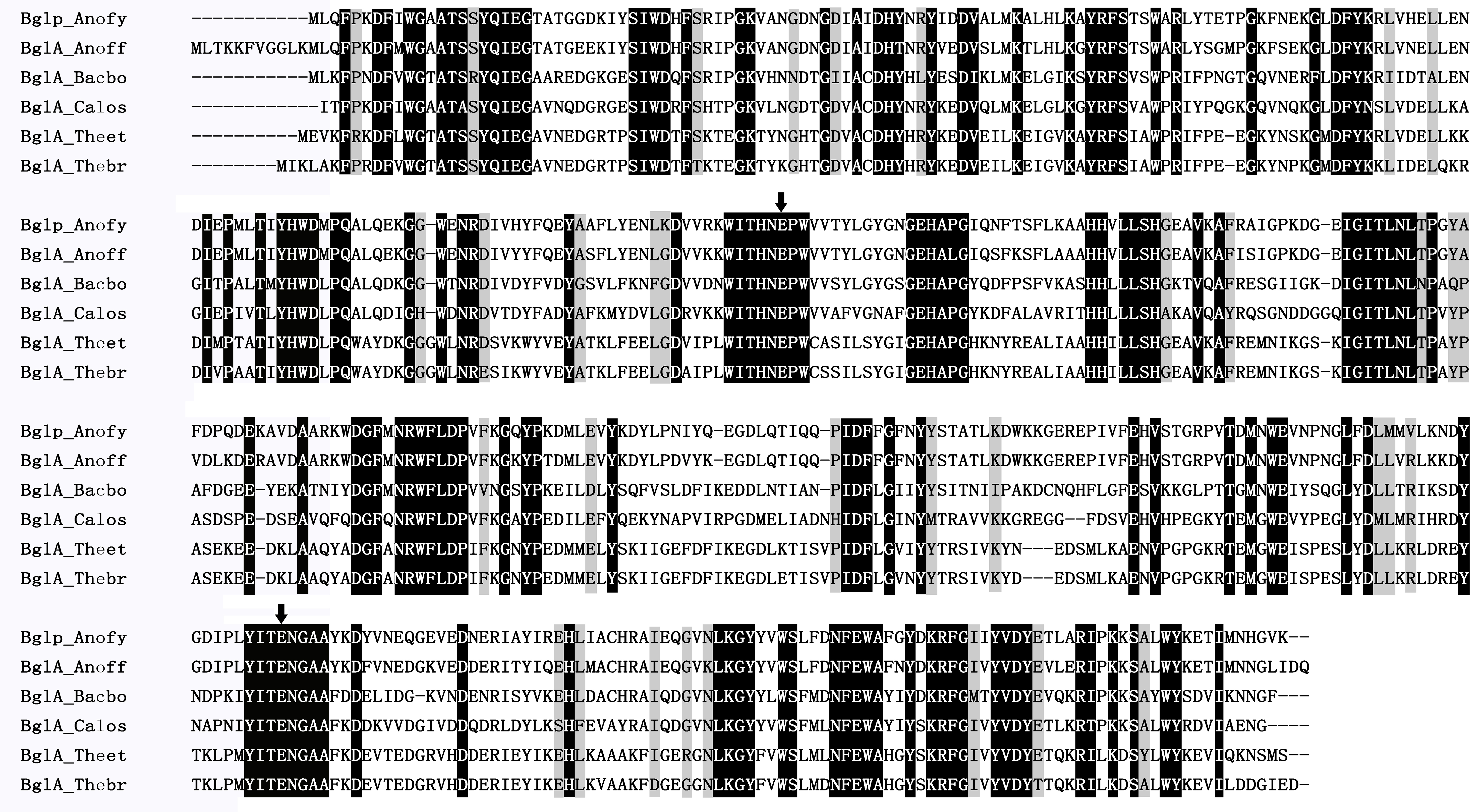


**Figure S1. Sequence alignment of Bglp with six similar β-glucosidases**. Full-length amino acid sequences were aligned by ClustalW. The identical residues are shown in white with a black background, and conservative changes are shown in black with a gray background. The two catalytic glutamate residues are indicated by black arrows. The amino acid sequences used were as follows. *Bglp_Anofy*, *A. flavithermus* subsp. *yunnanensis* E13^T^ (accession number KF453503); *BglA_Anoff*, *A. flavithermus* subsp. *flavithermus* WK1 (accession number ACJ34717); *BglA_Bacbo*, *Bacillus bogoriensis* (accession number WP_026675503); *BglA_Calos*, *Caldicoprobacter oshimai* (accession number WP_025747479); *BglA_Theet*, *Thermoanaerobacter ethanolicus* (accession number ADD25173); *BglA _Thebr*, *T. brockii* (accession number CAA91220).

3, SDS-PAGE analysis of recombinant BglP


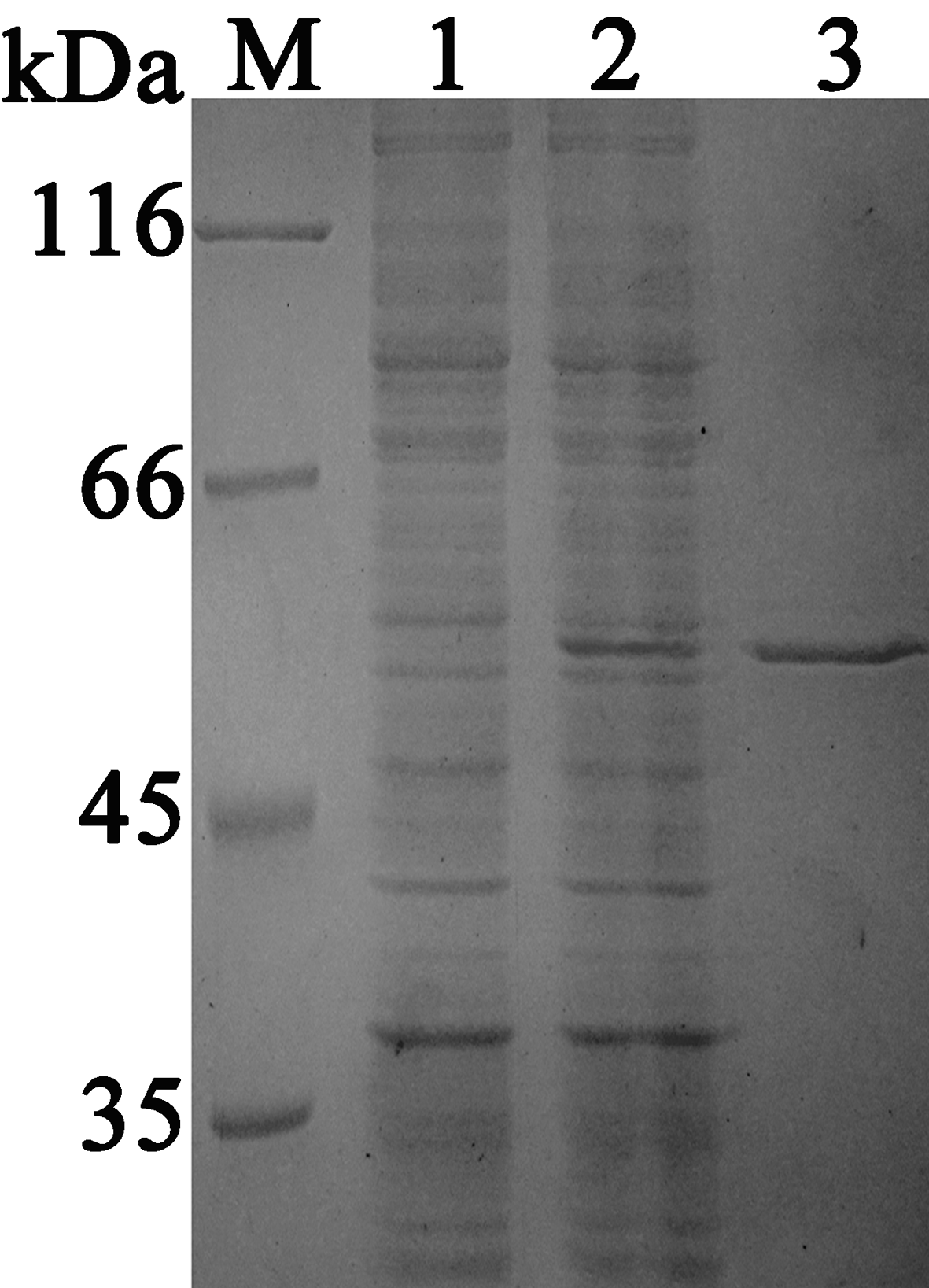


**Figure S2.** SDS-PAGE analysis of recombinant BglP. Lane M, protein marker; lane 1, cell extract of *E. coli* BL21(DE3) harboring pET22b; lane 2, cell extract of *E. coli* BL21 (DE3) harboring pET22b*-bglP*; lane 3, the purified BglP protein.

4, Kinetic Parameters


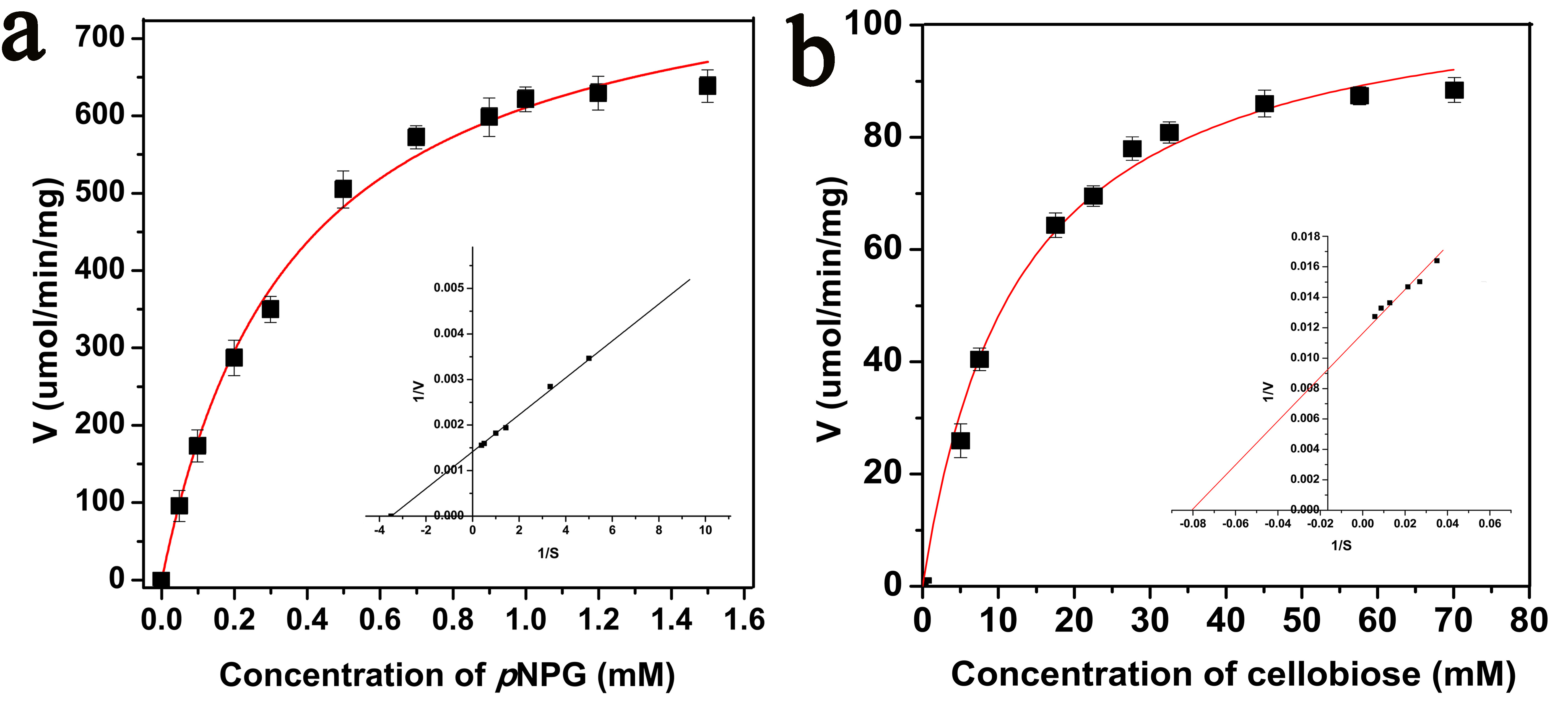


**Figure S3.** Effects of various concentrations of pNPG (a) and cellobiose (b) on the activity of recombinant BglP. Inset: Lineweaver-Burk plots. Each point represents the mean value of six determinations ± SD (Standard Deviation). These standard deviations were less than 10%.


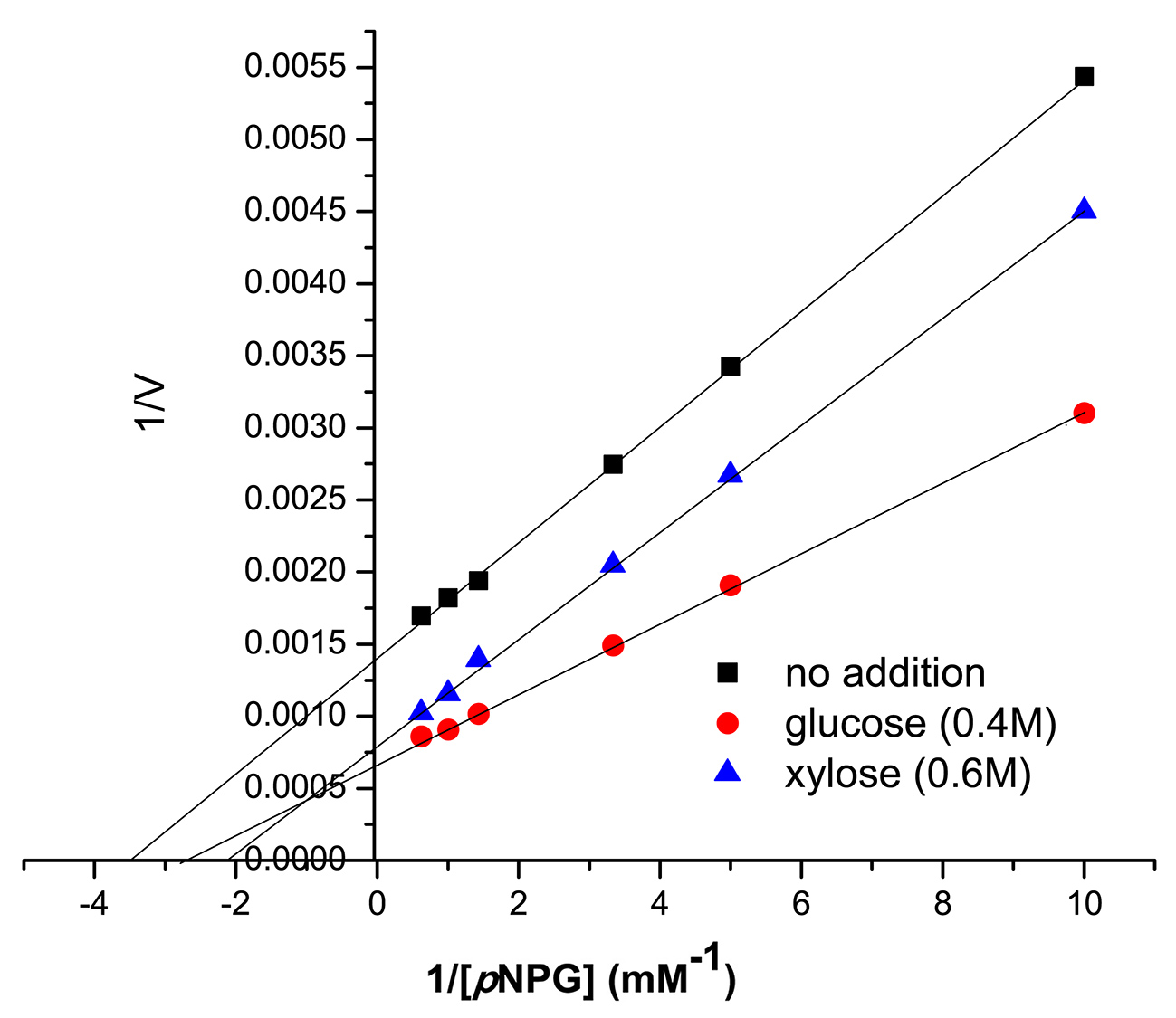


**Figure S4.** Lineweaver-Burk plots of *p*NPG hydrolysis by the recombinant BglP in the absence addition (■) and presence of fixed concentrations of glucose (●) or xylose (▲).

5, Inhibitory kinetics


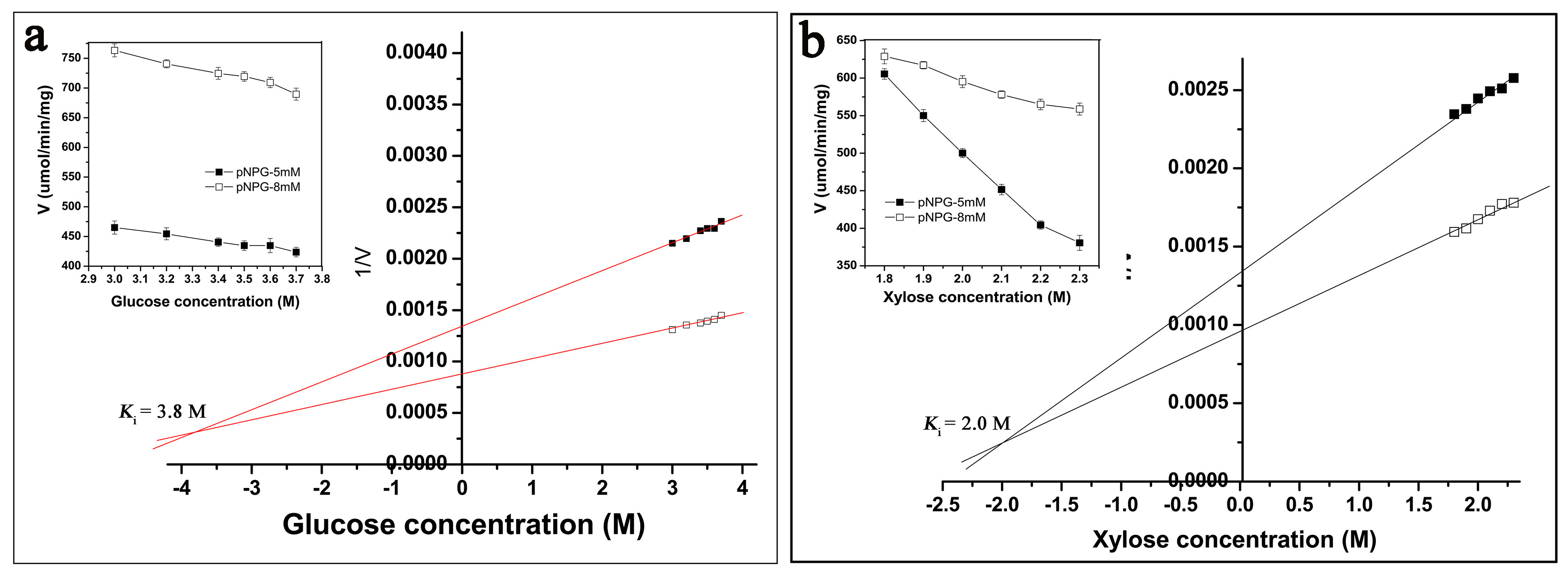


**Figure S5.** Dixon plot of inhibitory effects of glucose (a) and xylose (b) on *p*NPG hydrolysis by recombinant BglP. The *p*NPG concentrations used were 5 mM (■) and 8 mM (□). The inhibition constant (*K*_i_) were calculated to be 3.8 ± 0.22 and 2.0 ± 0.15 M for glucose and xylose, respectively. Inset: Effects of glucose and xylose on the hydrolysis rate.

6,

**Table S1** **Effect of sugar additions on the** **BglP activity****^a^**

| Additive | Relative activity (%) |
| --- | --- |
| None | 100.0 ± 0.5 |
| Glucose | 151.5 ± 1.6 |
| Xylose | 139.3 ± 1.2 |
| Galactose | 110.0 ± 0.9 |
| Arabinose | 108.3 ± 0.4 |
| Mannose | 103.7 ± 0.8 |
| Maltose | 101.4 ± 1.5 |
| Fructose | 101.2 ± 1.3 |
| Sucrose | 100.6 ± 0.7 |
| Cellobiose | 100.2 ± 1.1 |
| Ribose | 99.5 ± 0.8 |

^a^The activity was measured under the standard assay conditions in the presence of 100 mM sugars. The 100% relative activity corresponded to 842 ± 15.5 U/mg, estimated in same conditions without any additional sugar. Values are the means ± SD of six experiments (n = 6).
